# Evaluation of neuroretina following i.v. or intra‐CSF AAV9 gene replacement in mice with MPS IIIA, a childhood dementia

**DOI:** 10.1111/cns.14919

**Published:** 2024-08-09

**Authors:** Helen Beard, Leanne Winner, Andrew Shoubridge, Emma Parkinson‐Lawrence, Adeline A. Lau, Siti N. Mubarokah, Tabitha‐Rose Lance, Barbara King, William Scott, Marten F. Snel, Paul J. Trim, Kim M. Hemsley

**Affiliations:** ^1^ Childhood Dementia Research Group Flinders Health and Medical Research Institute College of Medicine and Public Health Flinders University Bedford Park South Australia Australia; ^2^ Healthy Microbiome and Chronic Disease, Lifelong Health Theme South Australian Health and Medical Research Institute (SAHMRI) Adelaide South Australia Australia; ^3^ Mechanisms in Cell Biology and Disease Research Group, Clinical Health Sciences UniSA Adelaide South Australia Australia; ^4^ Proteomics, Metabolomics and MS‐Imaging Facility South Australian Health and Medical Research Institute (SAHMRI) Adelaide South Australia Australia

**Keywords:** AAV9, gene therapy, mouse, mucopolysaccharidosis type III, photoreceptor degeneration, pre‐clinical, sulfamidase

## Abstract

**Background:**

Sanfilippo syndrome (mucopolysaccharidosis type IIIA; MPS IIIA) is a childhood dementia caused by inherited mutations in the sulfamidase gene. At present, there is no treatment and children with classical disease generally die in their late teens. Intravenous or intra‐cerebrospinal fluid (CSF) injection of AAV9‐gene replacement is being examined in human clinical trials; evaluation of the impact on brain disease is an intense focus; however, MPS IIIA patients also experience profound, progressive photoreceptor loss, leading to night blindness.

**Aim:**

To compare the relative efficacy of the two therapeutic approaches on retinal degeneration in MPS IIIA mice.

**Methods:**

Neonatal mice received i.v. or intra‐CSF AAV9‐sulfamidase or vehicle and after 20 weeks, biochemical and histological evaluation of neuroretina integrity was carried out.

**Results:**

Both treatments improved central retinal thickness; however, in peripheral retina, outer nuclear layer thickness and photoreceptor cell length were only significantly improved by i.v. gene replacement. Further, normalization of endo‐lysosomal compartment size and microglial morphology was only observed following intravenous gene delivery.

**Conclusions:**

Confirmatory studies are needed in adult mice; however, these data indicate that i.v. AAV9‐sulfamidase infusion leads to superior outcomes in neuroretina, and cerebrospinal fluid‐delivered AAV9 may need to be supplemented with another therapeutic approach for optimal patient quality of life.

## Graphical abstract

Beard, Hemsley and colleagues compared the efficacy of neonatal AAV9‐based gene replacement via intravenous or cerebrospinal fluid injection routes, on retinal degeneration in a mouse model of childhood dementia caused by Sanfilippo syndrome. Intravenous AAV9‐CMV‐human sulfamidase resulted in superior therapeutic efficacy, compared with cerebrospinal fluid infusions of the same viral vector. Created with BioRender.com.

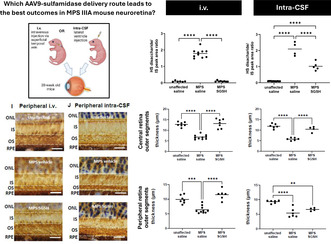

## 1 INTRODUCTION

Sanfilippo syndrome type A or mucopolysaccharidosis IIIA (MPS IIIA) is a childhood dementia‐causing disorder that is presently untreatable. It arises due to autosomal recessive mutations in the sulfamidase (*SGSH*) gene encoding a lysosomal sulfatase involved in the stepwise degradation of heparan sulfate (HS), leading to its accumulation in lysosomes, with cell dysfunction and death ensuing.^1^ Children with the classical form of the disease exhibit early and progressive cognitive impairment, sleep difficulties, autistic traits, motor dysfunction, and vision loss. The median age of death in children with rapidly progressing MPS IIIA is 18 years.^2^

Treatment is mostly supportive; however, a variety of potential therapeutics either have been or are currently being evaluated in human clinical trials. The most active areas of research are recombinant human enzyme e.g., Refs. [3, 4] or gene replacement, while substrate inhibition and targeting of the innate immune response are also being examined (www.clinicaltrials.gov NCT01299727; 02053064; 03300453; 03423186; 03612869; 04018755; 04088734). Viral vectors used for gene therapy in patients include AAVrh10‐human sulfamidase delivered via multisite intra‐cerebral infusion (NCT01474343; NCT02053064; NCT03612869),^5^, ^6^ scAAV9‐U1a‐sulfamidase delivered via intravenous (i.v.) injection (NCT02716246; NCT04088734; NCT04360265)^7^ and AAV9‐CAG‐coh‐sulfamidase delivered via intra‐ventricular cerebrospinal fluid (CSF) injection (EudraCT: 2015‐000359‐26).^8^, ^9^ The human clinical trials are ongoing, and no data has been published in the peer‐reviewed literature to date, to our knowledge. Further, direct comparison of the two AAV9‐sulfamidase delivery methods has not been undertaken to the best of our understanding; however, preclinical data indicate that both routes of injection lead to significant reductions in brain pathology and improved cognitive function in treated mice.^7^, ^8^

Less is known about the impact of the therapies on the retina, however. While the field is naturally focussed on preventing brain disease, MPS III patients exhibit profound and progressive lesions in neuroretina leading to loss of night vision,^10^, ^11^, ^12^, ^13^ findings that are replicated in animal models.^14^, ^15^, ^16^, ^17^ Significant early endo‐lysosomal expansion occurs in MPS IIIA mouse retina leading to changes in microglial morphology by 3–6 weeks of age.^14^ Loss (or reduction in length) of rod photoreceptor outer segments is noted in central MPS IIIA mouse retina by six weeks of age, but inner segment loss was not apparent until 20 weeks of age. Impaired electroretinogram recordings are evident from ~12 weeks of age in MPS IIIB mice,^15^ with progressive deterioration noted thereafter.

Improvements in retinal pathology were reported after i.v. AAV9‐sulfamidase delivered to one‐month old MPS IIIA mice by Fu et al.^7^ Whether intra‐CSF AAV9‐sulfamidase prevents or slows retinal disease lesion formation has not been reported to date. To enable a head‐to‐head comparison of the two clinically relevant routes of delivery of AAV9‐sulfamidase, we performed intra‐CSF infusions of vector into the lateral ventricles of neonatal MPS IIIA mice and the impact on retinal disease at 20 weeks of age was compared to that observed following i.v. delivery of the same vector at the same disease stage. Our rationale was that should the two putative therapeutic approaches prove equivalent in preventing/slowing cognitive decline in human clinical trials, their respective efficacy in nervous system tissues other than brain may identify one of the two routes of administration as a strategy of choice, given that the goal of therapy is to maximize both the quantity and quality of life of patients with MPS IIIA.

## 2 RESULTS

**FIGURE 1:**
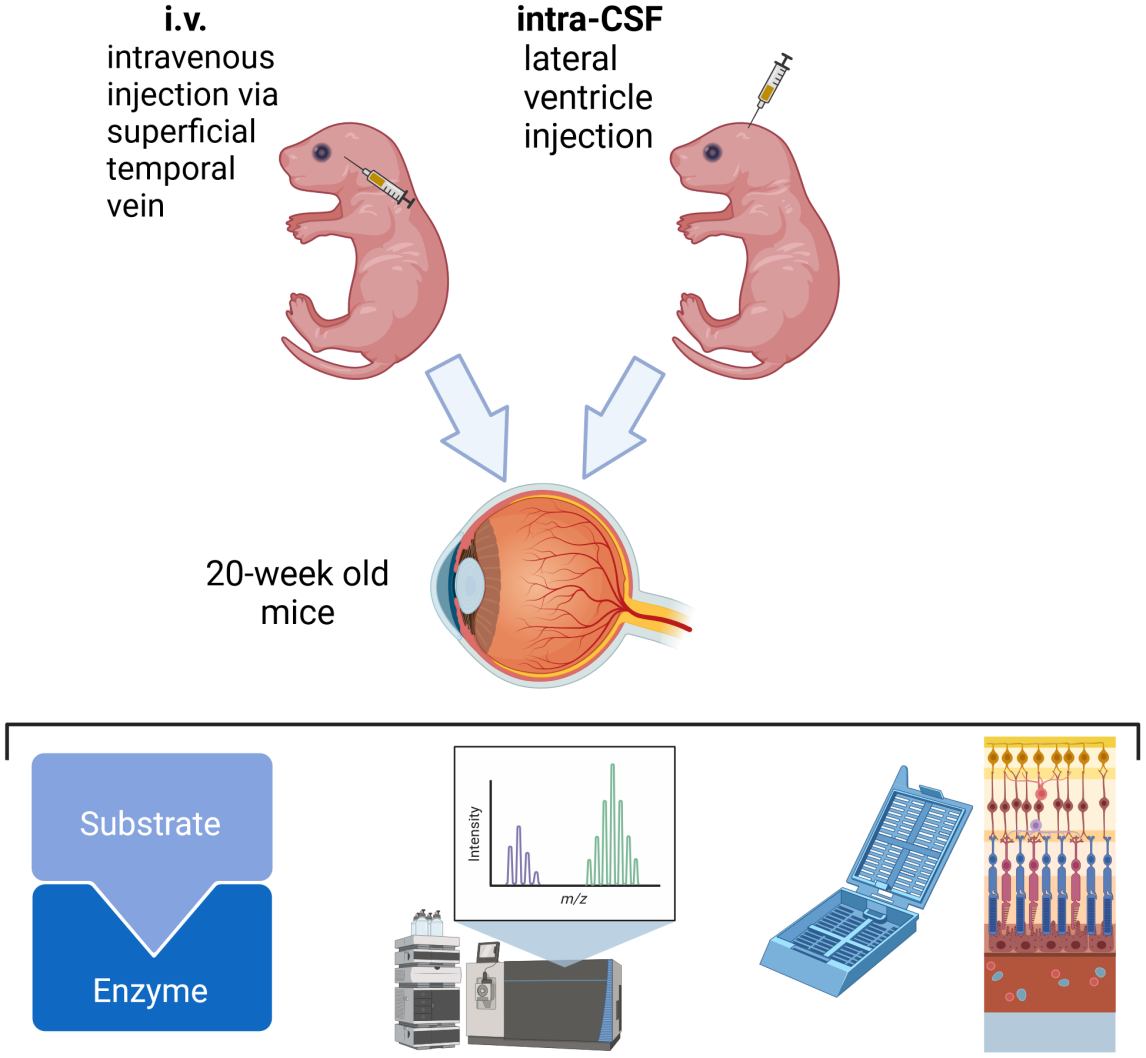
Overview of the study. Mice were injected on days 0–1 of life (*n* = 15–16/group). Injections were made intravenously (i.v.), via the superficial temporal vein or intra‐CSF, via the lateral ventricles. Mice treated with i.v. AAV9 received a second injection on day 5 of life. At 20 weeks of age, mice were euthanased and the eyes were removed and processed for quantification of sulfamidase activity, heparan sulfate accumulation, and for immunohistochemical or histochemical evaluation of the impact of treatment on neuroretina (*n* = 4–13/group). Created with BioRender.com

Groups of conscious male/female pups received treatment with the AAV9‐CMV‐human sulfamidase vector via injections made into the superficial temporal vein (referred to hereafter as i.v.) and tail vein. Infusions were also made into ventricular CSF (referred to hereafter as intra‐CSF) in additional cohorts of cryoanaesthetised pups. The study protocol is shown in Figure 1. Significant loss of MPS IIIA pups was observed following intra‐CSF infusions, regardless of infusate type (PBS as vehicle or vector). Of fifteen mice per group, only 5–6 per group remained at weaning. Death of wildtype vehicle‐treated mice also occurred, but to a lesser degree (13 of 15 mice survived to weaning). Litter loss has previously been reported in MPS IIIA mouse colonies^18^ and is increased when mice are disturbed.^19^ In the cohorts of mice/group receiving i.v. infusions, only one litter of six MPS IIIA mice that had been injected with PBS was cannibalized. As one MPS IIIA mouse eye from the intra‐CSF AAV9‐sulfamidase treatment group was damaged upon retrieval and not evaluated further, group sizes for analysis at 20 weeks of age varied from *n* = 4–13. Data from individual mice are presented throughout.

### 2.1 i.v. delivered AAV9‐sulfamidase normalizes endo‐lysosomal compartment size

**FIGURE 2:**
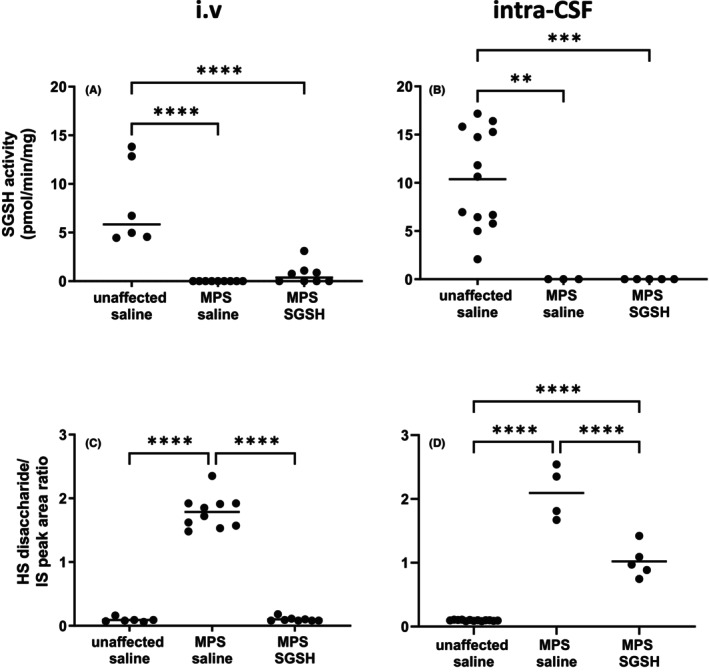
Effect of neonatal i.v. (A, C) or intra‐CSF (B, D) delivery of AAV9‐CMV‐sulfamidase on sulfamidase activity (A, B) and HS accumulation (C, D) in 20‐week‐old MPS IIIA mouse retina. IS = internal standard. **p < 0.01, ***p < 0.001, ****p < 0.0001. Only statistically significantly different comparisons between groups are shown.

**FIGURE 3:**
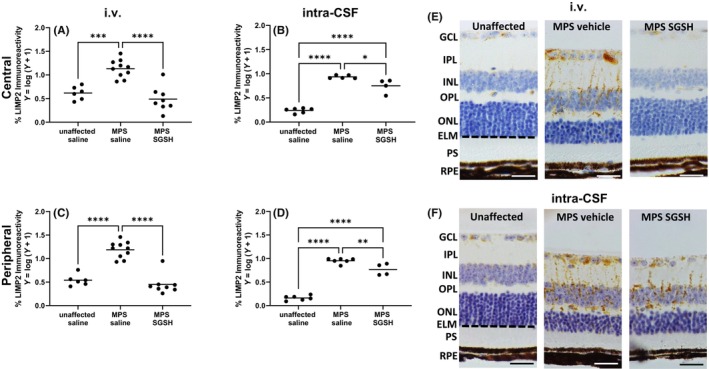
Effect of neonatal i.v. or intra‐CSF delivery of AAV9‐CMV‐sulfamidase on endo‐lysosomal compartment size in 20‐week‐old MPS IIIA mouse retina. Lysosomal integral membrane protein 2 (LIMP2) staining from the GCL to the external limiting membrane was assessed in both central and peripheral retina (A–D). Representative photos demonstrating the staining in the peripheral retina are provided for i.v. (E) and intra‐CSF delivery (F). Ganglion cell layer (GCL), inner plexiform layer (IPL), inner nuclear layer (INL), outer plexiform layer (OPL), outer nuclear layer (ONL), external limiting membrane (ELM, dashed), photoreceptor segments (PS), retinal pigmented epithelium (RPE). Scale bar = 25 μm. *p < 0.05, **p < 0.01, ***p < 0.001, ****p < 0.0001. Only statistically significantly different comparisons between groups are shown.

As shown in Figure 2A,B, MPS IIIA mice exhibit greatly reduced sulfamidase activity in retina compared to wildtype and increases in enzyme activity were only seen in mice treated via i.v. AAV9‐sulfamidase. Intravenous infusion of vector normalized HS levels (Figure 2C), whereas significant amounts of HS remained in intra‐CSF AAV9‐sulfamidase treated MPS IIIA mouse retina (Figure 2D). Compatible with the HS outcomes, staining of the endo‐lysosomal compartment with antibodies to lysosomal integral membrane protein 2 (LIMP2; Figure 3) revealed that while intra‐CSF AAV9‐sulfamidase significantly reduced the size of the compartment, only i.v. AAV9‐sulfamidase treatment was able to normalize it. As expected based on previously published studies, i.v. AAV9‐sulfamidase treatment increased brain SGSH activity, but to a lower level than that seen following intra‐CSF infusion of AAV9‐sulfamidase (18.6% of unaffected vs. 97.7% of unaffected SGSH activity, respectively; Figure S1). These observations are compatible with the relative amount of hSGSH mRNA transcripts that were detectable in brain following treatment via each route, with ~22× more transcript recorded in the brain of mice treated via intra‐CSF infusion (Figure S1). Treatment with AAV9‐sulfamidase via both routes completely normalized HS levels in brain.^20^, ^21^

### 2.2 Impact of the two treatments on the integrity of individual retinal cell layers

**FIGURE 4:**
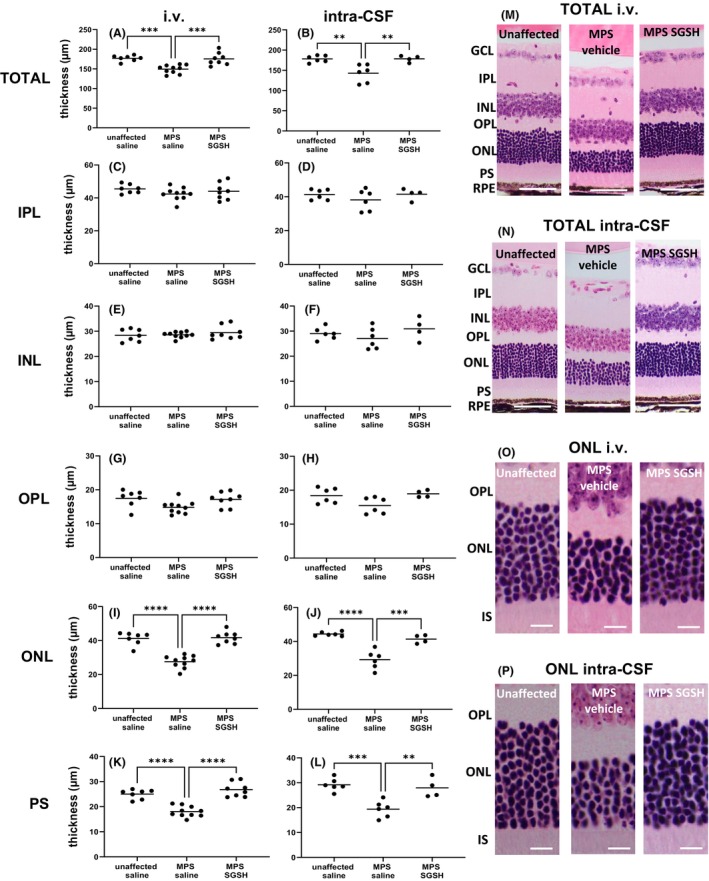
Effect of neonatal i.v. or intra‐CSF delivery of AAV9‐CMV‐sulfamidase (or vehicle) on central retinal thickness in 20‐week‐old mice. The thickness of total retina from the GCL to but not including the RPE was measured in H&E‐stained sections (A, B), as was the thickness of individual retinal layers (C–L). Representative photos show total retina in i.v. or intra‐CSF treated mice (M, N respectively) or ONL only (O, P respectively). Ganglion cell layer (GCL), inner plexiform layer (IPL), inner nuclear layer (INL), outer plexiform layer (OPL), outer nuclear layer (ONL), photoreceptor segments (PS), inner segments (IS), and retinal pigmented epithelium (RPE). Scale bar in M, N is 50 μm. Scale bar in O, P is 10 μm. **p < 0.01, ***p < 0.001, ****p < 0.0001. Only statistically significantly different comparisons between groups are shown.

**FIGURE 5:**
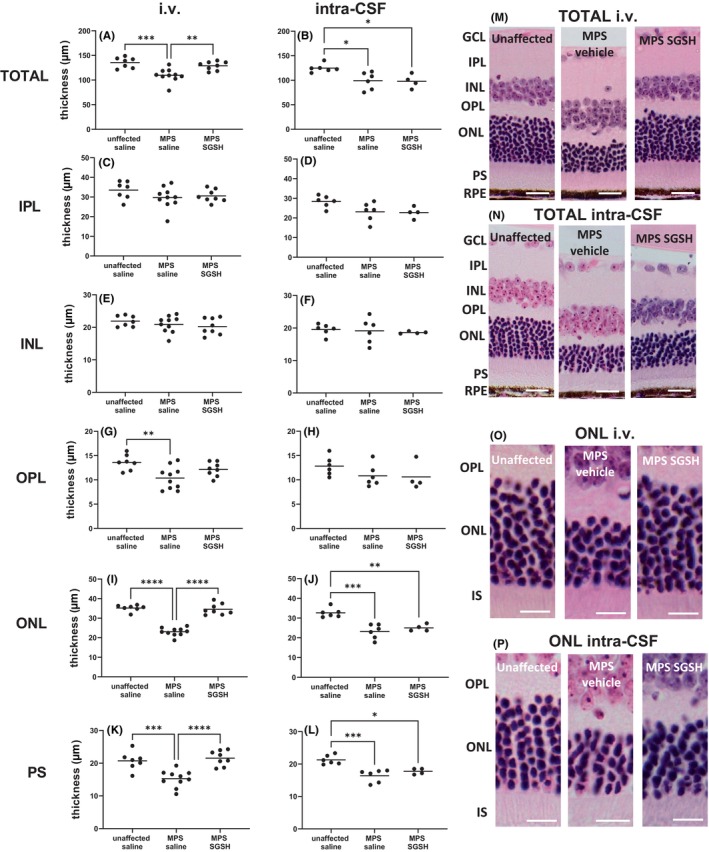
Effect of neonatal i.v. or intra‐CSF delivery of AAV9‐CMV‐sulfamidase (or vehicle) on peripheral retinal thickness in 20‐week‐old mice. The thickness of the retina from the GCL to but not including the RPE was quantified in H&E‐stained sections (A, B). The thickness of individual retinal layers was also evaluated (C–L). Photos of the total retina (M, N) or ONL (O, P) in representative mice from each treatment group are shown. Ganglion cell layer (GCL), inner plexiform layer (IPL), inner nuclear layer (INL), outer plexiform layer (OPL), outer nuclear layer (ONL), photoreceptor segments (PS), inner segments (IS), and retinal pigmented epithelium (RPE). Scale bar in M, N is 20 μm. Scale bar in O, P is 10 μm. *p < 0.05, **p < 0.01, ***p < 0.001, ****p < 0.0001. Only statistically significantly different comparisons between groups are shown.

As shown in Figure 4, total retinal thickness nearer the optic nerve (central) is decreased in MPS IIIA mice, mostly due to outer nuclear layer (ONL) thinning following photoreceptor segment (PS) loss. Both treatments prevented loss of photoreceptors in this region of retina, and the thickness of the retinal layers was not different from that seen in control/unaffected mice. In contrast, when measurements were taken further away from the optic nerve (peripheral; Figure 5), photoreceptor loss was unable to be prevented by intra‐CSF AAV9‐sulfamidase but was completely prevented by i.v. AAV9‐sulfamidase.

### 2.3 i.v. but not intra‐CSF AAV9‐sulfamidase leads to retention of rhodopsin‐positive photoreceptors across retina

**FIGURE 6:**
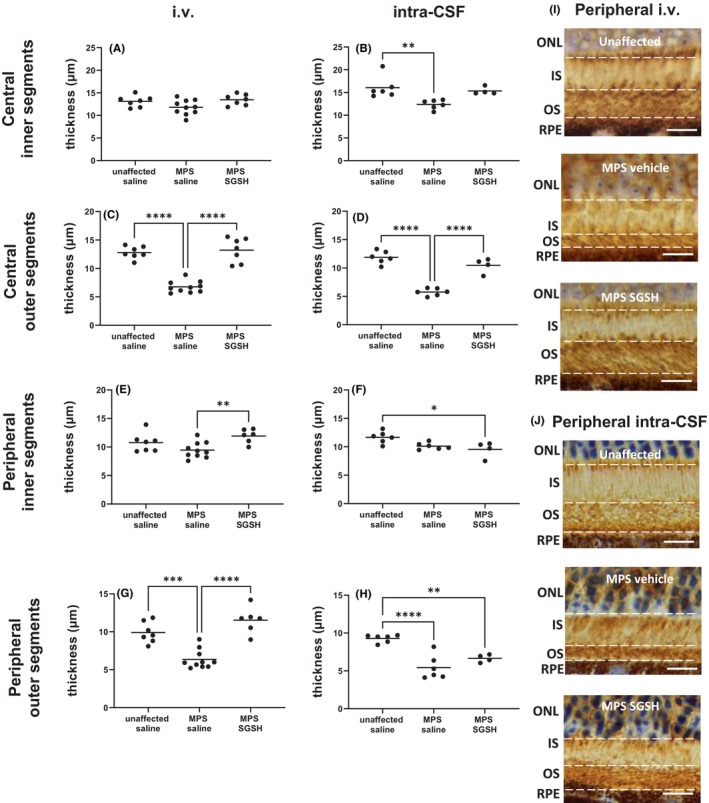
Effect of neonatal i.v. (A, C, E, G) or intra‐CSF delivery (B, D, F, H) of AAV9‐CMV‐sulfamidase (or vehicle) on rhodopsin‐positive photoreceptor IS (A–B, E–F) and OS (C–D, G–H) in the central (A–D) and peripheral (E–H) aspects of retina in 20‐week‐old mice. Representative photos of rhodopsin staining in peripheral retina of i.v. (I) and intra‐CSF AAV9‐treated mice (J) are shown. Outer nuclear layer (ONL), inner segments (IS), outer segments (OS), retinal pigmented epithelium (RPE). Scale bar in I, J is 10 μm. *p < 0.05, **p < 0.01, ***p < 0.001, ****p < 0.0001. Only statistically significantly different comparisons between groups are shown.

Rhodopsin staining of rod photoreceptors revealed that outer segment (vs. inner segment) loss is pronounced in the retina of MPS IIIA mice (Figure 6) and was prevented in central retina by gene replacement delivered via both routes. Consistent with the above results, only i.v. AAV9‐sulfamidase was able to prevent the loss of outer segments in peripheral retina.

### 2.4 There are fewer isolectin‐B4‐reactive microglia in retinae of mice treated with i.v. AAV9 versus intra‐CSF AAV9

**FIGURE 7:**
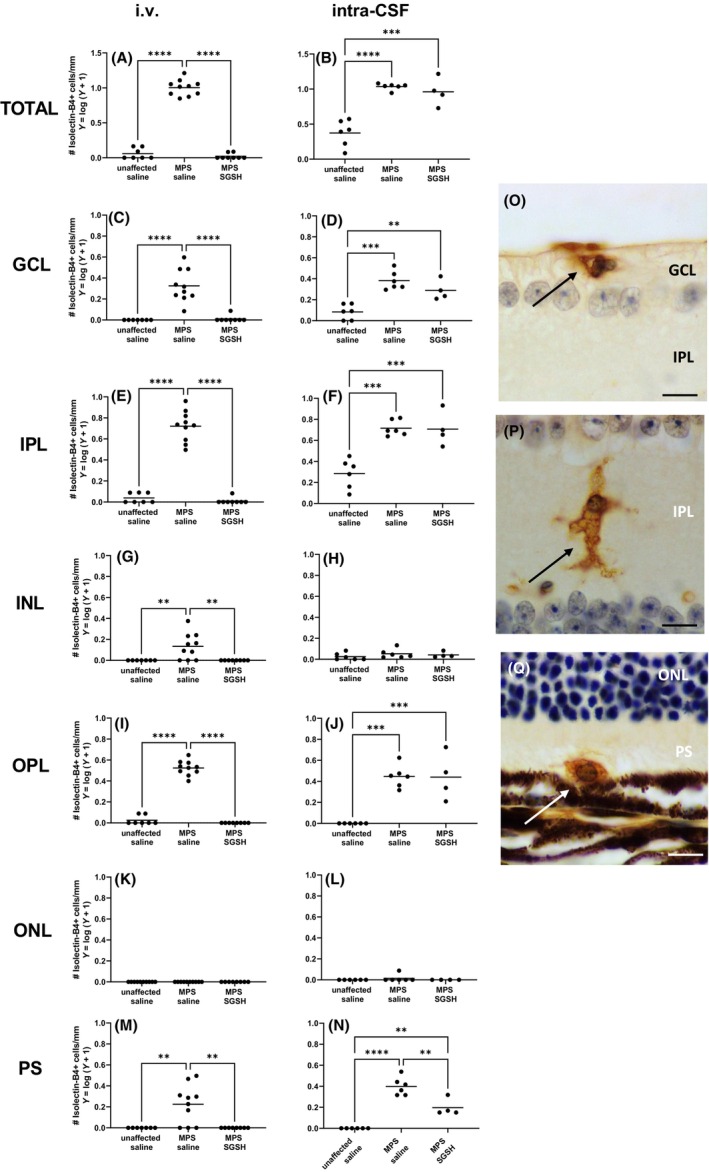
Impact of AAV9‐sulfamidase (or vehicle) delivered i.v. (A, C, E, G, I, K, M) or intra‐CSF (B, D, F, H, J, L, N) on the number of ameboid isolectin B4‐positive cells per mm of retina in 20‐week‐old mice. Counts were performed along the whole length of the retina. Each retinal layer was assessed. Representative photos of stained/counted microglia (arrows) are shown in; ganglion cell layer (GCL; O) inner plexiform layer (IPL; P), photoreceptor segment (PS; Q). Inner plexiform layer (IPL), inner nuclear layer (INL), outer plexiform layer (OPL), outer nuclear layer (ONL), photoreceptor segments (PS). Scale bar in O–Q is 10 μm. **p < 0.01, ***p < 0.001, ****p < 0.0001. Only statistically significantly different comparisons between groups are shown.

Large numbers of isolectin B4‐positive microglia with an ameboid morphology are noted in 20‐week‐old MPS IIIA mouse retina (Figure 7). i.v. AAV9‐sulfamidase prevented development of these morphological changes in all retinal layers. In contrast, intra‐CSF AAV9‐sulfamidase was only able to partially improve the microglial phenotype in the photoreceptor layer and similar numbers of ameboid isolectin‐B4‐reactive microglia were seen in the GCL, IPL, and OPL of intra‐CSF vehicle‐ and intra‐CSF AAV9‐sulfamidase‐treated mice.

## 3 DISCUSSION

This study sought to compare the ability of two clinically relevant viral vector delivery approaches in preventing retinal disease in MPS IIIA mice. We have previously demonstrated that the AAV9‐based gene therapy strategies used here normalize HS in MPS IIIA mouse brain at 20 weeks (Shoubridge, PhD Thesis).^21^ Previous studies have also demonstrated that retinal degeneration in murine MPS IIIA begins early—at around six‐weeks of age, with significant and progressive loss of photoreceptor outer segments.^14^ Elevated levels of HS and the appearance of ameboid‐shaped isolectin‐B4‐positive microglia are detectable by three‐weeks of age^14^; thus, in the present proof‐of‐principle study, we sought to prevent retinal degeneration by providing human sulfamidase gene replacement to mice at birth. We also note that MPS IIIA patient clinical trials have recently begun restricting entry to infants under 24 months (e.g., NCT04201405, NCT02716246), thus treatment of very young subjects is appropriate.

We used a single vector design—single‐strand AAV9‐CMV‐human sulfamidase but treated some mice via intravenous infusion and other mice via intra‐ventricular CSF injection. These two delivery routes are currently being examined as strategies for providing AAV9‐based gene therapy to children with MPS IIIA (NCT02716246 and EudraCT 2015‐000359‐26, respectively). Given that most of the vector genomes end up in the liver following i.v. infusion (e.g., Ref. [7]) and only 5%–10% of vector genomes are detected in brain, we gave larger amounts of vector to mice receiving i.v. infusion. Our dosing strategy is also compatible with the range of doses provided clinically. Providing equivalent vector genomes i.v. and intra‐CSF would undoubtably lead to either vector‐mediated side effects, e.g., reactive gliosis in brain if the dose given intra‐CSF was larger to match that provided i.v., or insufficient vector reaching central nervous system if the dose given i.v. was lower to match that given intra‐CSF. As expected, despite the disparity in vector dose, much lower amounts of hSGSH transcript and SGSH activity were detected in brain following i.v. versus intra‐CSF infusion of AAV9‐SGSH.

While both injection routes facilitate sulfamidase delivery to central retina, for the first time we demonstrate that i.v. but not intra‐CSF infusions of AAV9‐CMV‐sulfamidase enable treatment of peripheral retina in MPS IIIA, a site distant from the optic nerve. In support of this, central retina versus medial or peripheral retina, was preferentially treated when mice with CLN6 deficiency which causes late infantile Batten disease, received intra‐CSF AAV9‐based gene replacement.^22^ Limited gene/protein expression in retina following injection of AAV vectors into the CSF has also been observed previously in additional models of Batten disease—sheep with CLN5 deficiency^23^ and dogs with TPP1 deficiency.^24^, ^25^

As higher vector dose does not explain this outcome based on the brain data, we hypothesize that the localized treatment of retina mediated by intra‐CSF injection of vector is due to the way the AAV9 particles or potentially the brain‐expressed human sulfamidase reaches the retina—via retrograde transport in ganglion cell axons in the optic nerve. AAV9 has been shown to undergo bidirectional transport in neurons, with retrograde transport occurring in a dynein‐dependent way.^26^, ^27^ Interestingly, intravitreal injection of AAV2‐β‐glucuronidase enabled this lysosomal enzyme to be transported anterogradely along the optic nerve into select murine brain regions.^28^ Vector genomes were not detectable in the brain of AAV2‐β‐glucuronidase‐treated mice. Thus, it is possible that the human sulfamidase produced in brain following intra‐CSF injection of AAV9‐sulfamidase has been transported retrogradely in retinal ganglion cell axons to the cell bodies in the retina, along with vector particles—but the treatment effect is limited to regions proximal to optic nerve transection, annotated as “central retina” in our study.

Systemically delivered AAV9, in contrast, has been shown to cross the neonatal mouse blood–retinal barrier via transcytosis,^29^ and has also been documented to access adult mouse retina following i.v. infusion.^30^ While studies in older animals are required for confirmation, if the outcomes in humans are comparable to those made here and elsewhere in mice, sheep, and dogs,^22^, ^23^, ^24^, ^25^ i.v. delivery of AAV9 may be the route of choice as it appears to enable most if not all neural and non‐neural tissues to be treated (Fu et al.^7^; this study). In contrast, Haurigot et al.^8^ and the abovementioned Batten disease model studies have shown that intra‐CSF infusion of AAV9‐based gene replacement leads to transduction of brain, spinal cord, and peripheral nervous system, e.g., peripheral ganglia, together with other organs; however, there is inferior access to, and thus treatment of, retina. Thus, another strategy is likely to be required for treating retinal abnormalities in patients treated with intra‐CSF‐directed gene replacement, for instance direct intraocular gene therapy. This resulted in significant improvements in retinal structure and function as demonstrated in MPS VII mice^31^ and CLN5‐deficient sheep.^32^

Finally, it is important to note that there are differences between the vector used here (AAV9‐CMV‐human sulfamidase) and the vectors being administered in trials of i.v. and intra‐CSF AAV9‐sulfamidase in children with MPS IIIA, that may affect the degree of retinal transduction and the speed and amount of gene expression. First, the promotor in the vector being administered in the clinical trial of i.v. gene therapy is a U1a promoter, rather than the CMV promoter used here. This constitutive small nuclear RNA promoter (U1a) appears to enable persistent high‐level gene expression in a pan‐cellular fashion.^33^ Further, the small size of this promotor is compatible with the smaller packaging capacity of self‐complementary AAV vectors such as that used in the i.v. gene therapy trial in MPS IIIA patients. Use of an scAAV9 vector may also hasten gene expression (e.g., Ref. [34]) compared to single‐strand AAV9 vectors like the one used here.

The promotor being used in the clinical trial of intra‐CSF infusion of single strand AAV9‐sulfamidase is CAG (cytomegalovirus early enhancer/chicken β‐actin). CAG and CMV promotors resulted in similar levels of reporter gene expression in brain following intra‐CSF infusion of AAV9 vectors to mice,^35^ although superior spinal cord expression of eGFP was noted with a CMV promotor. Finally, the vector in the intra‐CSF infusion clinical trial contains a codon‐optimized human sulfamidase gene. Codon optimization was not carried out here, nor has it been utilized to our knowledge in the i.v. AAV9‐sulfamidase clinical trial. Codon optimization is performed to remove potentially deleterious unmethylated CpGs in cDNA that initiate immune stimulation leading to elimination of transduced cells.

In summary, our findings indicate that intra‐CSF viral vector‐based treatment of MPS IIIA patients will potentially need to be supplemented with an intraocular therapeutic to modify the profound and progressive retinal dysfunction seen in this disorder. Patients receiving i.v. AAV9‐sulfamidase may not need supplementary treatment of retinal disease if these findings in neonatal mice are translatable to young children. Evaluation and reporting of whether retinal dysfunction is halted or prevented in children treated with i.v. AAV9‐sulfamidase (or not) is urgently needed. While the improvement or maintenance of cognitive function is of supreme importance when assessing the efficacy of a therapeutic in MPS IIIA patients, for maximum quality of life, the prevention of vision loss also requires increased focus.

## 4 MATERIALS AND METHODS

### 4.1 Approvals

This research protocol was approved by the Institutional Animal Ethics (approvals #1109/12/21 and #1073/11/2020) and Biosafety Committees (#B144/12/2020 and #B149‐12‐21) prior to study commencement.

### 4.2 Vector

The single strand AAV9‐CMV‐human sulfamidase vector was synthesized by Vector Labs (Malvern, PA USA) and provided at a concentration of 9.2 × 10^13^ genome copies per mL.

### 4.3 Mice

The mice used in this study were sourced from breeding colonies maintained at the Women's and Children's Hospital Network, North Adelaide, South Australia, Australia. Congenic MPS IIIA mice (B6.Cg‐Sgsh^mps3a^) and congenic MPS IIIA mice crossed with a reporter strain (B6.Cg‐Tg(Thy1‐YFP)HJrs/J; JAX Stock #003782) were used. All mice were group‐housed in a temperature/humidity‐controlled facility with a 14‐h light: 10‐h dark cycle. Food and water were available ad libitum. The mice received toilet rolls and plastic cups plus nesting material for enrichment purposes. All breeding, housing, and experimental procedures complied with the Association for Research in Vision and Ophthalmology (ARVO) statement and the Australian code for the care and use of animals for scientific purposes (8th edition; 2013).

### 4.4 Genotyping

The presence/absence of the D31N mutation in the murine *Sgsh* gene was determined according to Lau et al. (2013).^36^ *YFP* copy number to determine null, hemizygous, or homozygous status was determined using a custom assay with the following primers: forward (5′ GCA CCA CCG GCA AGC T 3′), reverse (5′ AGT CGT GCT GCT TCA TGT GGT 3′), and YFP probe (5′ FAM‐ACC ACC TTC GGC TAC G‐NFQ‐MGB 3′). Mouse Tfrc was used as the reference gene (VIC‐labeled #4458366; ThermoFisher Scientific, Waltham, MA, USA). Sequences were amplified as follows: 2 min at 50°C, 10 min at 95°C, 40 cycles of PCR amplification (15 s at 95°C followed by 1‐min at 60°C) with 10 ng genomic DNA per well, using 384‐well plates (#4309849; ThermoFisher Scientific). QuantStudio Real‐Time PCR and CopyCaller v2.1 software were used for data analysis (ThermoFisher Scientific).

### 4.5 Mouse injections

i.v. injections were made into awake pups via the superficial temporal vein on day 1 and the tail vein on day 5 of life (Figure 1). On each occasion, 5 × 10^11^ vector genomes were delivered in 10 μL (dosing strategy as per Byrne et al.^29^). Intraventricular CSF injections were given on day 0 of life to cryoanaesthetised pups. Briefly, this involved cryoanaesthesia of mice followed by transillumination of the head using a MicroLight 150 Cold Light Fiber Optic Illuminator (Fibreoptic Lightguides, VIC, Australia) and infusion of vector through a 27G dental needle held in the arm of a stereotaxic frame (David Kopf Instruments, CA, USA). The needle was attached to polyethylene tubing (Becton Dickinson, USA), connected to a Hamilton syringe which was placed on a slow infusion pump (SP200iz Syringe Infusion Pump (World Precision Instruments, Sarasota, FL, USA)), permitting controlled infusion of vector (1 μL/min). Injections were made at the mid‐point between lambda and bregma, 1 mm lateral to the sagittal suture and 2 mm deep. Mice recovered under a warm lamp. A total of 5 × 10^10^ vector genomes was delivered in 2 μL (1 μL per hemisphere; dose as per Haurigot et al.^8^). The twenty‐fold difference in vector genomes delivered accounts for the fact that only ~5%–10% of vector genomes reach the central nervous system (i.e., brain) compared with liver following i.v. injection.^7^

The mice were genotyped via ear notching at ~2 weeks of life and weaned at three weeks as per usual husbandry procedures. When they reached 20 weeks of age, mice were humanely euthanised using CO_2_ overdose. Intra‐cardiac perfusion with cold PBS was followed by enucleation and immersion fixation of eyes in Davidson's fixative overnight. Brain and some eyes were removed and stored frozen at −80°C.

### 4.6 Sulfamidase activity and heparan sulfate assays

Retinae from one eye were placed in Lysing Matrix D tubes (MP Biomedicals) and homogenized in 0.02 M Tris/0.5 M NaCl, pH 7.4, using a Precellys 24 Tissue Homogenizer. Homogenates were then dialyzed into 0.2 M sodium acetate buffer pH 6.5 overnight at 4°C. Brain samples were directly homogenized in 0.5 mL of 0.2 M NaAc pH 6.5. Sulfamidase activity was determined using a 4‐MU‐based fluorimetry assay according to previously published methods,^37^ with data reported as pmol/min/mg of total protein. Total heparan sulfate‐derived disaccharides were then quantified using acid hydrolysis followed by tandem mass spectrometry, a previously published method.^38^ Peak area ratios were determined using Analyst 1.6.2 software (ABSciex, Concord, Ontario, Canada) and data are expressed per mg of total protein. Total protein was quantitated using a MicroBCA kit (#23235, ThermoFisher Scientific).

### 4.7 RNA isolation and RT‐qPCR for transgene expression analysis

There was insufficient retinal tissue to assess hSGSH transcript levels. Total RNA was extracted from brain homogenates using TRIzol™ reagent according to manufacturer protocol and treated with DNaseI (Life Technologies). Two micrograms of RNA was reverse transcribed to cDNA with random primers using a High‐Capacity cDNA Reverse Transcription Kit (Life Technologies). Detection and quantification of human SGSH transgene expression was performed by real‐time PCR using a Bio‐Rad CFX Opus 384 Real‐Time PCR system with SsoAdvanced™ Universal SYBR® Green Supermix (Bio‐Rad). Cycles were 98°C for 30 s, 40 cycles of 98°C for 15 s and 60°C for 1 min. The primer sequences used to detect human SGSH were: forward primer 5′‐CTCTTTCGCAATGCCTTCAC‐3′, reverse primer 5′‐TGTCGAAGGAGTTGAAGTGG‐3′. The housekeeping gene used was mouse β‐actin: forward primer 5′‐GGTCATCACTATTGGCAACG‐3′, reverse primer 5′‐ACGGATGTCAACGTCACACT‐3′. Data were normalized to the mRNA abundance of the housekeeping gene β‐actin and are presented as comparative Ct values, using 2^−ΔCt^ quantification.^39^

### 4.8 Immunohistochemistry and histochemistry

The other eye from each mouse was fixed in Davidson's solution (2‐parts 37% formaldehyde, 3‐parts 100% ethanol, 1‐part glacial acetic acid, 3‐parts water) for 24 h and was then processed into paraffin. Six‐micron thick section sections were cut using a rotary microtome (Leica, Wetzlar, Germany) in a consistent manner to the level of the optic nerve head. Sections were mounted on glass slides (Superfrost™ Plus, Thermo Scientific, USA), oven dried and rehydrated after xylene deparaffinization and ethanol washing. Hematoxylin and eosin (H&E) staining was performed using standard methods. For immunohistochemical and histochemical staining of retina, antigen retrieval pretreatment, primary antibody and isolectin B4 histochemical staining reagents used are outlined in Table S1.

Immunohistochemical staining was performed according to established methods.^14^ Briefly, antigen retrieval was performed prior to blocking nonspecific labeling with 10% normal donkey serum (NDS, #017‐000‐121, Jackson ImmunoReasearch Laboratories, PA, USA) in PBS. Sections were incubated overnight in primary antibody diluted in 2% NDS, washed in PBS, and then endogenous peroxidases were blocked with 0.3% hydrogen peroxide. Sections were incubated at room temperature in species‐specific biotinylated secondary antibody, 1:2000 (Jackson ImmunoResearch Labs, PA, USA), followed by Vectastain ABC reagent (#PK 6100: Vector Laboratories, CA, USA). Color detection was achieved with the diaminobenzidine (DAB) liquid chromogen system (#3468: DAKO, Glostrup, Denmark). To detect ameboid, presumptively activated microglia, sections underwent antigen retrieval and were then incubated in 0.3% hydrogen peroxide followed by peroxidase‐conjugated isolectin‐B4 overnight. Color detection was achieved with DAB.

Staining was batched, and all analysis was conducted by a user blinded to mouse genotype and treatment status. Sections were viewed on either an Olympus BX41 (with an Olympus UC50 camera), or an OlympusBH‐2 microscope (with an Olympus DP22 camera).

### 4.9 Quantification of retinal thickness

H&E‐stained sections were evaluated to determine total retinal thickness and individual retinal layer thickness. Measurements were taken in two locations: 500 μm from the optic nerve head (representing “central” retina) and 500 μm from the ciliary body (representing “peripheral” retina), to ensure inter‐animal consistency. Measurements were made on each side of the optic nerve head, and the mean thickness of the peripheral and central retina was calculated. Retinal thickness was only determined in sections in a perpendicular orientation. Total thickness represents the distance from the retinal ganglion cell layer (RGC) to, but not including the retinal pigmented epithelium (RPE). The thickness of the inner plexiform layer (IPL), inner nuclear layer (INL), outer plexiform layer (OPL), outer nuclear layer (ONL), and total photoreceptor segment layer (PS) was also determined. Rhodopsin‐stained sections were used to measure the inner segment (IS) and outer segment (OS) thickness. All measurements were carried out blind to genotype/treatment group.

### 4.10 Quantification of immunohistochemical labelling

Threshold analysis of % LIMP2 positive immunoreactivity was performed using FIJI image analysis software^40^ on images taken using a 40× objective from the ganglion cell layer to the external limiting membrane (ELM), in the central and peripheral retina. The number of amoeboid isolectin B4‐positive cells was counted manually along the entire length of the retina, with individual retinal layers assessed. Counts were reported as number of amoeboid microglia/mm.

### 4.11 Statistical analysis

Individual data points are shown, and the group mean is indicated. Graphical and analytical exploratory data analyses were performed (including normality which was examined using the Shapiro–Wilk test, given the small n in some groups), and patterns assessed. Analyses were performed on log‐transformed data for LIMP‐II and isolectin B4‐stained retinae to satisfy normality assumptions. Multiple comparisons were handled with ANOVA and *post hoc* Bonferroni correction (GraphPad Prism version 8). Data were regarded as statistically significant when *p* < 0.05. *p < 0.05, ** *p* < 0.01, ***p < 0.001, ****p < 0.0001.

## AUTHOR CONTRIBUTIONS

KMH conceptualized the study, HB, LW, AAL, SNM, BK, TRL, and WS undertook formal data analysis, KMH acquired the funding, KMH, HB, LW, AS, AAL, SNM, BK, TRL, WS, and PJT carried out the investigation, KMH, HB, AAL, SNM, MFS, and PJT developed the methods used and KMH and HB prepared the data for presentation. EJP‐L co‐supervised AS, a PhD candidate at the time this study was performed and contributed intellectually to the design of the intra‐CSF portion of the study. KMH administered the project, supervised the study, and wrote the original draft. All authors reviewed and edited the manuscript.

## CONFLICT OF INTEREST STATEMENT

KMH has received funding from Shire Human Genetic Therapies and Lysogene, for pre‐clinical evaluation of enzyme replacement and AAVrh10‐based gene therapy (respectively) for Sanfilippo syndrome. No authors are affiliated with or have received funding from any organization associated with the two human clinical trials of AAV9‐sulfamidase in patients with Sanfilippo syndrome.

## Supporting information


Figure S1.

Table S1.

## Data Availability

The data that support the findings of this study are available from the corresponding author upon reasonable request.
