# Supplementary material for: Evaluation of neuroretina following i.v. or intra‐CSF AAV9 gene replacement in mice with MPS IIIA, a childhood dementia
Source: CNS Neurosci Ther. 2024 Aug 9;30(8):e14919. doi: 10.1111/cns.14919 (PMC11315678; doi:10.1111/cns.14919)
Supplement: Supplementary file 1 — Figure S1. [file CNS-30-e14919-s001.docx]

**Supplementary Figure 1** – AAV-derived hSGSH gene expression (A, B) and SGSH activity (C, D) in brain homogenates. **p<0.01, ****p<0.0001. hSGSH gene expression was unable to be determined in retina due to insufficient material. ND = not detected.

**intra-CSF**

**i.v.**

**B**

**A**

**C**

**D**
