# Supplementary material for: Evaluation of neuroretina following i.v. or intra‐CSF AAV9 gene replacement in mice with MPS IIIA, a childhood dementia
Source: CNS Neurosci Ther. 2024 Aug 9;30(8):e14919. doi: 10.1111/cns.14919 (PMC11315678; doi:10.1111/cns.14919)
Supplement: Supplementary file 2 — Table S1. [file CNS-30-e14919-s002.docx]

**Supplementary Table 1:** Details of reagents and conditions used for immunohistochemistry and histochemistry.

| *Primary antibody/ Lectin* | *Catalogue number, Source* | *Dilution* | *Pre-treatment* |
| --- | --- | --- | --- |
| Mouse anti-rhodopsin | #MAB5316; Sigma-Aldrich Pty. Ltd. NSW, Australia | 1:600 | 10 mM citrate, 2 mM EDTA, 0.5% Tween 20, pH 6.0, 15-min microwave |
| Mouse anti-lysosomal integral membrane protein 2 (LIMP2) | In-house; Hemsley et. al., (2008) | 1:500 | 1mM EDTA, 0.05% Tween 20, pH 8.0  15-minutes microwave |
| Isolectin-B4-peroxidase conjugated | #L5391; Sigma-Aldrich Pty. Ltd. NSW, Australia | 1:60 | 0.05% Trypsin, pH 7.6  15 minutes @ 37°C |
